# Sequential electron beam and bioflocculation for treatment of textile nanodyes

**DOI:** 10.1039/d3ra03895e

**Published:** 2023-07-19

**Authors:** Nora M. Elkenawy, Ola M. Gomaa

**Affiliations:** a Drug Radiation Research Department, National Center for Radiation Research and Technology (NCRRT), Egyptian Atomic Energy Authority (EAEA) Cairo Egypt; b Radiation Microbiology Department, National Center for Radiation Research and Technology (NCRRT), Egyptian Atomic Energy Authority (EAEA) Cairo Egypt olagomaa4@gmail.com

## Abstract

Nanodyes are a new class of hazardous materials that are used in textile coloring. Their small size, color, stability and high dispersion characteristics pose a huge threat if they are released in open water systems. The aim of the present study is to test electron beam irradiation, bioflocculation and their sequential use for nanodye removal. The nanodye was obtained from a factory and was characterized using UV-visible spectroscopy, Fourier transform infra-red (FTIR) spectroscopy, dynamic light scattering, zeta potential and energy dispersive X-ray (EDX). The obtained results show that applying 7.5 kGy electron beam irradiation results in complete color removal in 10 min for 50 and 100 ppm nanodye, while at 200 and 400 ppm concentrations, the decolorization reaches 90% but leaving a residual brownish color. Adding 5 mg mL^−1^ of *Serratia marcescens* N2 biosurfactant resulted in agglomeration of 80% dye removal for 400 ppm nanodye after 24 h. On the other hand, the use of sequential electron beam and bioflocculation led to an initial removal of 80% in 1 h. The residual dyes were tested for toxicity on normal dermal HFB4 cells. The toxicity result was 1.19% after electron beam treatment, while those for sequential treatment and bioflocculation were 6.28 and 6.9%, respectively. It can be concluded that electron beam technology provides fast and highly efficient nanodye removal, while biosurfactants offer a low-cost, eco-friendly approach with a chance for dye retrieval.

## Abstract 2

Nanodye removal strategy depends on initial dye concentration.

## Graphical abstract

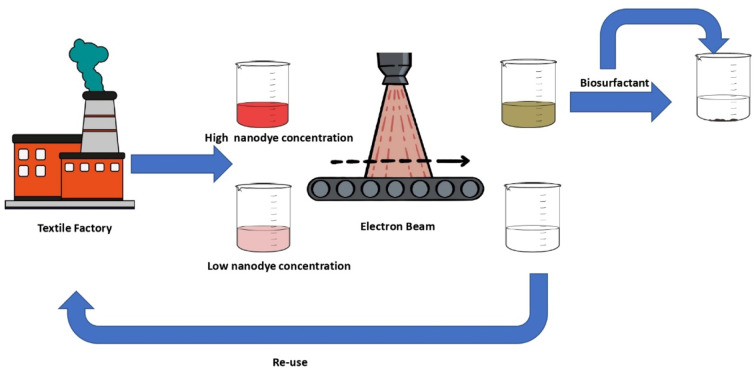

## 1. Introduction

There is a huge global water scarcity problem that is expected to affect world agriculture and health especially since the general direction is to reuse wastewater as a new water resource.^1^ Therefore, wastewater treatment should be very efficient to ensure the highest quality of treated wastewater. Industrial wastewater has been suggested as one of the options to replenish the difference between water availability and usage due to water scarcity problems.^2^ The surplus of generated wastewater prompted its use as a water resource after suitable treatment that ensures safety for use and according to the required application. For example, treated wastewater used for agriculture is different from that used for cleaning purposes.^3^ Textile wastewater for instance needs to be treated efficiently due to the myriad of complex organic compounds, heavy metals and salts that render it toxic.^4^

Textile industry is considered one of the main industries consuming water and generating wastewater.^5^ It is estimated that 5000 tons of dyestuffs are released into the environment each year causing different environmental and aesthetic problems.^6^ Azo dyes are a group of dyes widely used in paper printing, textiles, plastics and cosmetics. Toxicity of textile dyes has been reported before on bacteria, algae and protozoans.^7^ Its effect may lead to cytotoxicity and genotoxicity and treating the effluent efficiently can help alleviate this toxicity.^8^ The hazardous effect of wastewater re-uses lies in the pathway of their release. Industrial wastewater is collected with household and agriculture drainage water, mixed and then treated for re-use in irrigation. The treated wastewater still poses different health risks through consuming the treated wastewater irrigated crops. Direct consumption of crops directly irrigated with treated wastewater or indirect consumption through aquatic animals and animals can lead to skin diseases, osteoporosis, cholera, cognitive disorders and anemia.^9^ Industrial wastewater, for example, can be taken out of the combined wastewater if treated in the factory and reused.

The development of microfibers and nanofibers created a need for nanosized dyes to provide dyeing coverage, stability and overall improvement of dyeing quality.^10^ The presence of nanodyes is expected to increase the toxicity of generated wastewater, rendering it more complicated to treat.

There are different textile wastewater treatment approaches such as physical, chemical and biological treatment processes.^11^ Each approach has its drawbacks in terms of time, cost or generated sludge. One of the physical approaches for breaking down dyes is the use of ionizing radiation. Ionizing radiation using accelerated electrons is an example of advanced oxidation process where a hydroxyl radical attacks chemical compounds breaking their bonds. During this process, highly reactive intermediates (*e.g.* hydroxyl radicals) generated by water radiolysis are the primary oxidants in the reaction, leading to the oxidation of organic dye degradation.^12–14^ The required dose for treatment varies according to the nature of the contaminant and the targeted application. Dye degradation, for example, can increase with increasing irradiation dose, its effect is attributed to the fact that at higher radiation dose, more reactive species are generated in solution, which in turn are available to react with and degrade dye molecules.^15^ The doses can be as low as 5 kGy^16^ and can reach 100 kGy.^17^ The degradation of the dyestuffs starts, exclusively, by the attack of OH˙ in places rich in electrons from the dyestuff molecules. The radical OH˙ formed is the main reactive kind that degrades the dyestuff, destroying or changing the structure of its chromophore group. Ionizing radiation from electron beams (EB) was discovered as an alternative to the advanced oxidation process for effluent treatment *via* important reactive types for the oxidation of organic contaminants.^18,19^ EB irradiation depends on irradiation materials or products *via* an electron beam that is generated by an electron accelerator. This high energy technology is simple and safe.^20^ EB irradiation and types of accelerators are reviewed by ref. 21.

Biological degradation of azo dyes may involve different mechanisms such as non-specific reductive process through redox-mediators, breaking of azo bonds by azoreductase enzymes and bacterial adsorption or bioflocculation of the dyes using biosurfactants.^22^ Biosurfactants are considered better than chemical surfactants since they have lower or negligible toxicity, and higher stability over a wide range of pH, temperature, and salinity. They are produced during microbial growth and are classified based on their chemical structure which are lipopeptides, glycopeptides, phospholipids and biopolymeric surfactants. They are reported to be produced by different bacteria, for example; *Pseudomonas taiwanensis*,^22^*Rhodococcus erythropolis*,^23^*Kurthia gibsonii*,^24^*Pseudomoas aeruginosa*,^25^*Serratia marcescens* N2 (ref. 26) and *Bacillus* sp.^27^ Their use in bioremediation was successfully reported for the removal of heavy metals and polyaromatic hydrocarbons, dyes^28^ and crude oil.^29^

A combined approach of both physical and biological treatment processes is expected to efficiently remove dyes.^27,30–32^ It was also reported that this combined approach can both remove the dyes and lower the toxicity.^33^ It can be a plausible way of achieving the maximal treatment with minimal drawbacks generated from a single treatment process. In the present study, electron beam irradiation and bioflocculation were tested as single and sequential approaches to obtain an efficient, non-hazardous and eco-friendly approach that would be suitable for re-use.^34^ The present work is the first to discuss the removal of nanodyes from aqueous media.

## 2. Materials and methods

### 2.1. Nanodye solution preparation

The nanodye used in the present study was obtained from a local textile factory located 118.7 km outside Cairo (30° 58′ 7′′ N, 31° 9′ 49′′ E). A 400 ppm stock solution was prepared from the obtained dye and stored in a brown bottle in the dark. The stock solution was used to prepare all the nanodye concentrations in the upcoming experiments.

### 2.2. Nanodye characterization

#### 2.2.1. Spectrophotometer

A UV-visible scan was performed for each sample. The scan was performed from 200–800 nm using spectrophotometer (SPECORD 210 plus, analytic Jena). Decolorization was calculated according to the following equation:
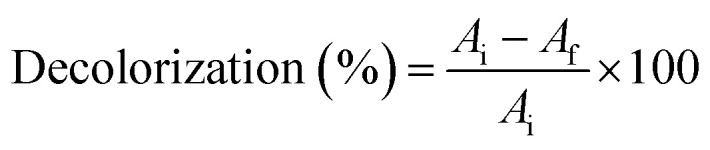
where *A*_i_ is the initial dye absorbance and *A*_f_ is the final dye absorbance. Dye concentrations were calculated from a standard curve prepared from different concentrations of nanodye at the nanodye absorbance maxima. The linear equation was *y* = 0.008*x* + 0.2258 and *R*^2^ = 0.9773.

#### 2.2.2. DLS

The mean droplet size of the nanodye at concentration was determined by the dynamic light scattering technique (DLS) employed by a PSSNICOMP Zeta Potential/Particle Sizer 380ZLS (PSS-NICOMP, Santa Barbara, CA, USA) at 25 °C with an average of 10 runs was calculated.

#### 2.2.3. Zeta potential

The net charge of the nanodye was obtained using PSSNICOMP Zeta Potential/Particle Sizer 380ZLS (PSS-NICOMP, Santa Barbara, CA, USA).

#### 2.2.4. Fourier transform infrared spectroscopy (FT-IR)

The identification of functional groups was carried out using Attenuated total reflectance-Fourier transform infrared spectroscopy (ATR-FTIR). Nanodye in its powder form was scanned within the range of 4000–400 cm^−1^ using BRUKER VERTEX 70 device at NCRRT. The spectrum obtained was compared to literature to identify the main functional groups.

#### 2.2.5. Energy dispersive X-ray (EDX)

About 10 mg of the nanodye powder was placed on a metal stub using double-sided adhesive tape. Elements were measured using EDX attached to scanning electron microscope (ZEISS-EVO 15-UK) operated at 25 keV. The analysis was performed using the full area.

### 2.3. Treatment of nanodye using electron beam irradiation

Electron beam irradiation was performed using glass vials with 1 mm thickness. The sealed vials contained 3 mL nanodye at concentrations (50, 100, 200 and 400 ppm) were exposed to electron beam irradiation at doses of (2.5, 5, 7.5 and 10 kGy) using an industrial linear accelerator, Beam 3 mA, from VIVIRAD, France, at the National Center for Radiation Research and Technology (NCRRT), Cairo, Egypt. The following parameters were applied: 3 MeV, conveyer speed 16 m min^−1^, providing a minimum dose of 3 kGy with no maximum dose. Readings were done in triplicates.

### 2.4. Treatment of nanodye using *Serratia marcescens* N2 culture and its culture filtrate

*Serratia marcescens* N2 used in the following experiment was previously isolated and identified Bioproject ID PRJNA525074, Biosample ID SAMN11041520, and WGS accession SPSG00000000.^35^ A 24 h *Serratia marcescens* N2 culture and its cell free culture filtrate was tested for nanodye decolorization. About 5% of nanodye stock solution was added to molten Luria Bertani agar media to reach final dye concentration of 50 ppm. The nanodye solution was sterilized using 0.2 μm membrane filter. The molten agar was quickly swirled to ensure homogeneity and dispersion of color in the agar media. The agar was poured into Petri dishes and a loopful of *Serratia marcescens* N2 was streaked on agar surface. The plates were left to incubate at 30 °C and were monitored to detect any color change. Color change was indicated as positive or negative. To test the bacterial culture filtrate, 1 mL of 7 day old *Serratia marcescens* N2 cell free culture filtrate was added to 50 ppm nanodye stock solution to test their capability of nanodye decolorization. Biosurfactant was produced as described in a previous study.^26^*Serratia marcescens* N2 was cultivated in 20% fish frying oil, 1% peptone and an inoculum concentration of 8% at 25 °C for 13 days.

### 2.5. Biosurfactant effect on different nanodye concentrations

About 5 mg mL^−1^ biosurfactant was added to 50, 100, 200 and 400 ppm nanodye. The color removal was monitored after 1, 3 and 24 h using spectrophotometric scanning. Decolorization was calculated as mentioned earlier. Preliminary screening experiment was done to select the chosen time points. Readings were done in triplicates.

### 2.6. Sequential treatment of both electron beam and bioflocculation

Sequential treatment of both electron beam and bioflocculation was performed by (1) exposing the 400 ppm dye to 7.5 kGy radiation using electron beam, (2) adding the bioflocculant to the 400 ppm dye, and (3) adding the bioflocculant to the electron beam irradiated dye as a second step. The color removal was monitored after 1, 3 and 24 h using spectrophotometric scanning. Decolorization was calculated as mentioned earlier. Readings were done in triplicates.

### 2.7. Cytotoxicity assays

Cytotoxicity effect of water treated using electron beam and bioflocculation and combined treatment against dermal skin cells HFB4 cells was done using method of functional assay of viability and cytotoxicity (MTT).^36–38^

### 2.8. Statistical analysis

Statistical analysis was done using Graphpad prism 9.5.730 software program. Two-way analysis of variance (ANOVA) test followed by Bonferroni's post test to compare multiple means by row. One-way analysis of variance (ANOVA) test followed by Tukey–Kramer multiple comparison's test.

## 3. Results and discussion

### 3.1. Nanodye characterization

**Fig. 1:**
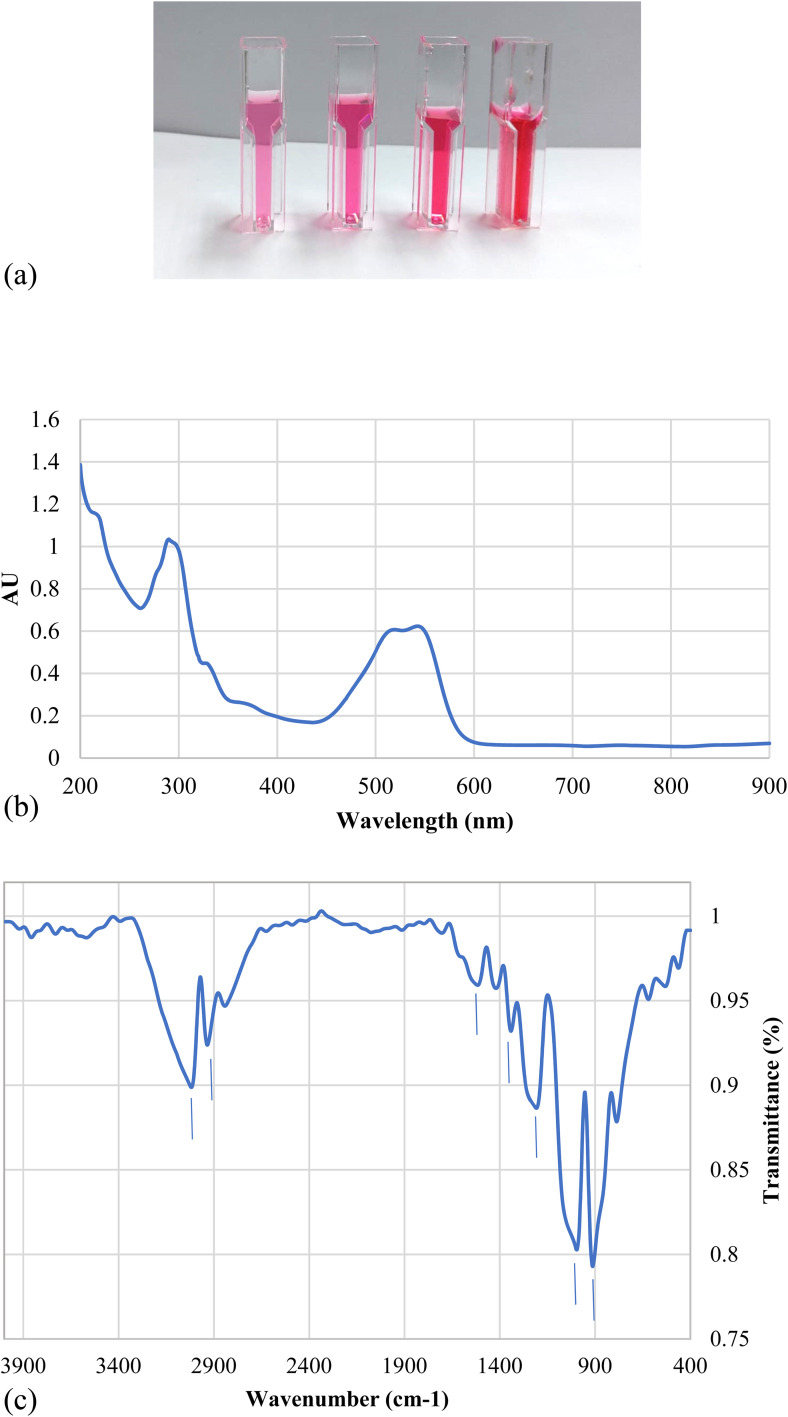
(a) Visible color for 50, 100, 200 and 400 ppm concentrations of the nanodye under study. (b) UV-visible spectrum for 50 ppm concentration of the nanodye under study. (c) FTIR spectrum for 50 ppm concentration of the nanodye under study.

**Fig. 2:**
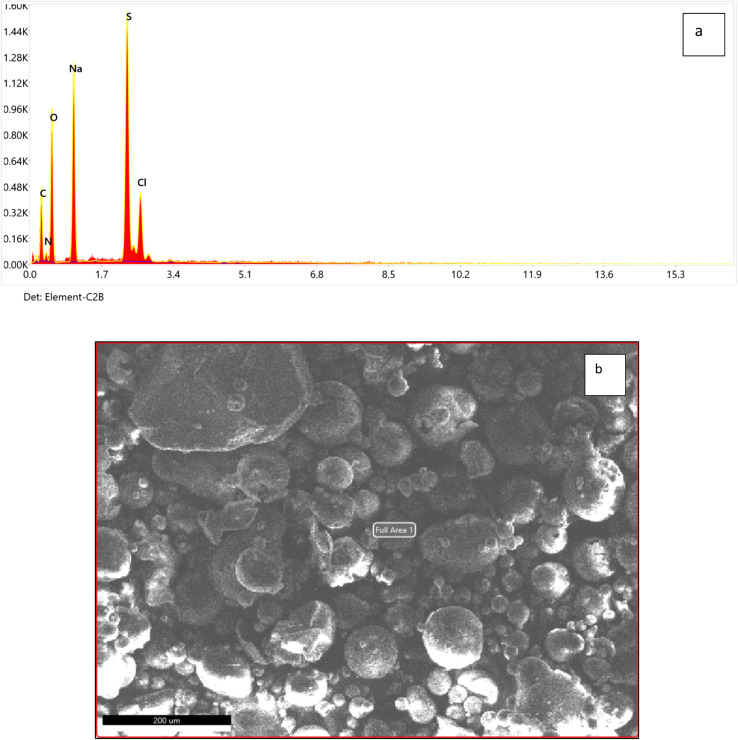
Elemental analysis of nanodye using EDX full area (a) and SEM image of the area for elemental analysis (b).

Nanodye characterization was done to identify the commercially obtained textile dye in the current study. The dye showed a dark pink to red color visually (Fig. 1a), and it showed distinctive peaks at 515 and 545 nm (Fig. 1b), which were reported as characteristic of red dyes.^39^ Peak at around 510 nm is characteristic of azo bond.^40^ Dynamic light scattering test of the dye showed that its size was 240 nm which confirms that it is of nano size.^41^ The surface charge of −28.58 mV and the pH of the dye is water soluble (Table 1), and its aqueous solution was pH 5 which indicates that this is an anionic dye. This result is in agreement with ref. 42 who stated that nanoparticles with zeta potential less than −30 mV are strongly anionic. Acid dyes were reported to be used on substrates such as wool, nylon, silk, inks, leather, and paper and have chemical structure of anthraquinone, xanthene, azo, nitro or triphenylmethane.^43^ FTIR spectrum (Fig. 1c) for the nano dye under study showed peaks at 3024.6 cm^−1^ and 2929.7 for =C–H (aromatic ring) and –OH stretching (carboxylic acid), respectively, 28 839.1 cm^−1^ for –CH_2_– symmetrical stretching, 1523.7 cm^−1^ for –N=N– stretching vibration, 1425 and 1342.4 cm^−1^ for aromatic –C=C– stretching and –C–N– aromatic stretching vibration, 1211.25 and 997 were for R-SO_3_^−^ asymmetric stretching vibration.^40^ The elemental analysis shows that the dye contains C, N, O and S along with Na and Cl (Fig. 2 and Table 2) which confirms that this dye is an azo dye that contains carboxylic and sulfate groups and its present in its sodium salt form.

**Table 1:** Some characteristics of the nanodye

Parameter	Value
Color	Red
Size (DLS) nm	235
Charge (zeta potential) mV	−28.58
Absorbance maxima (*λ*)	515, 545
pH	5

**Table 2:** Elemental analysis of nanodye using EDX-SEM

Element	Weight%	Atomic%
C K	32.15	43.16
N K	4.49	5.16
O K	32.94	33.19
Na K	16.88	11.84
S K	10.12	5.09
Cl K	3.42	1.56

### 3.2. Electron beam irradiation effect on nanodye

**Fig. 3:**
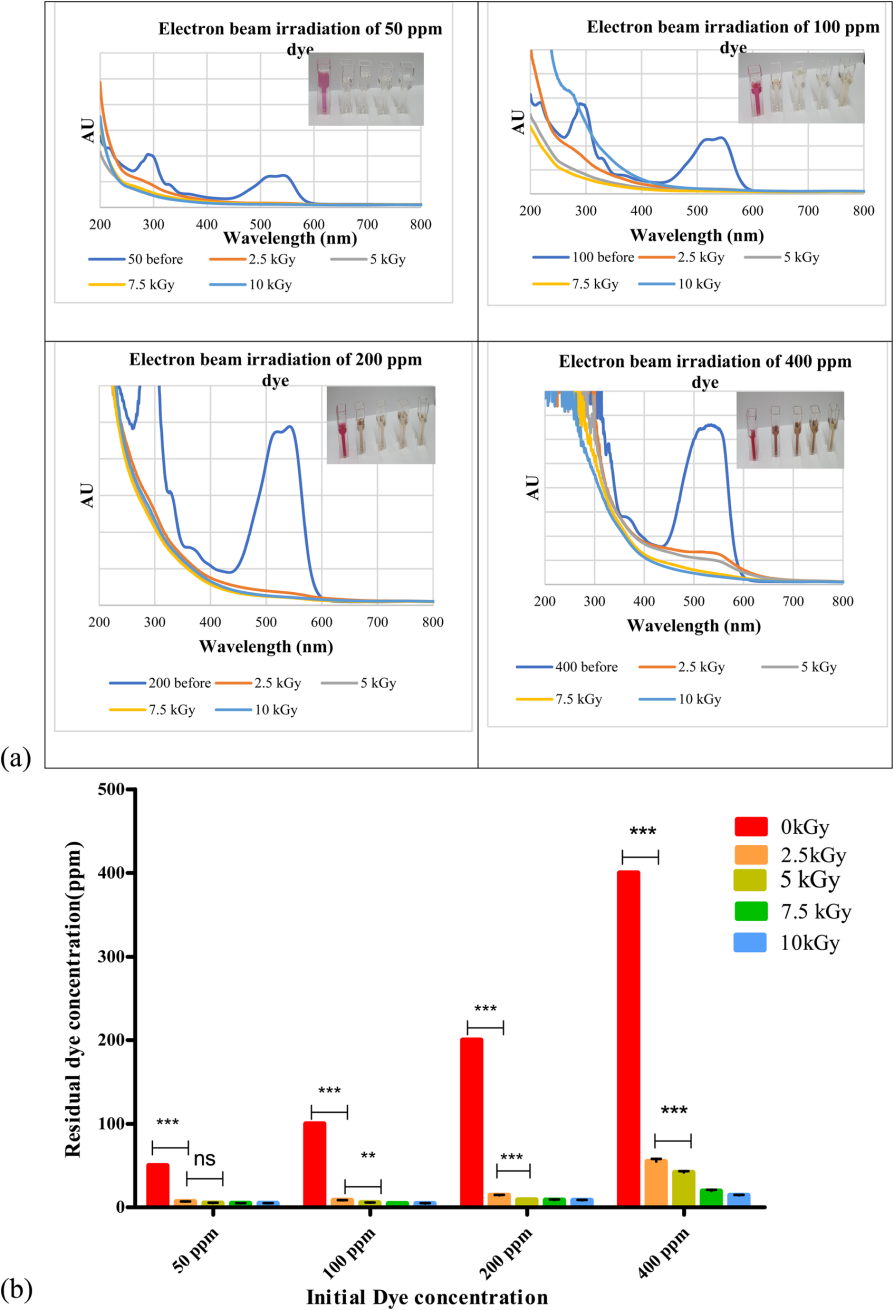
(a) Visible spectrum for dye concentration at 50, 100, 200 and 400 ppm nanodye after exposure to 0, 2.5, 5, 7.5 and 10 kGy electron beam irradiation. Inset pictures show the color change for each concentration. (b) Residual dye concentration for 50, 100, 200 and 400 ppm nanodye after exposure to 0, 2.5, 5, 7.5 and 10 kGy electron beam irradiation. Two-way analysis of variance (ANOVA) test followed by Bonferroni's post-test to compare multiple means by row.

Fig. 3a represents the change in spectrum in different nanodye concentrations when the dyes were exposed to 2.5, 5, 7.5 and 10 kGy of ionizing radiation at the electron beam facility. The spectra show complete decolorization of the dyes at concentrations 50 and 100 ppm, while a residual brownish color can be seen for concentrations of 200 and 400 ppm. This can be seen from the insert photos for each concentration. Fig. 3b shows that the decolorization is above 90% for all concentrations despite the residual brownish color for the high nanodye concentrations. This means that the chromophore group of this red nanodye has been destroyed. It is worthy to mention that the decolorization using electron beam irradiation takes only 10 min to be completed. Ionizing radiation by electron beam provides an advanced option to develop reactive oxidation process for the textile effluent treatment for the oxidation of organic contaminants through a series of chemical steps.^18^ It was noted that increasing the dose above 7.5 kGy didn't improve decolorization as expected as the brown colour remained.^44^ that the yield of decolorization decreased with increasing absorbed dose, since the rate constants of radicals' reactions with original dye molecules and with the products of dye degradation are of the same order; therefore, while the concentration of the latter increases during irradiation, the fraction of radicals in reactions with those products also increases, thereby reducing the fraction of radicals required for “decolorization” of the original dye molecules.^44^

### 3.3. *Serratia marcescens* N2 culture and culture filtrate for nanodye removal

As a pilot experiment, both *Serratia marcescens* N2 culture and its previously produced biosurfactant were tested for their ability to decolorize the nanodye under study. The preliminary results showed no decolorization when using the bacterial culture. This may be due to the vivid red color of *Serratia marcescens* N2 culture and culture filtrate^26^ that can interfere with the spectral analysis of the red nanodye under study.

### 3.4. Biosurfactant effect on different nanodye concentrations

**Fig. 4:**
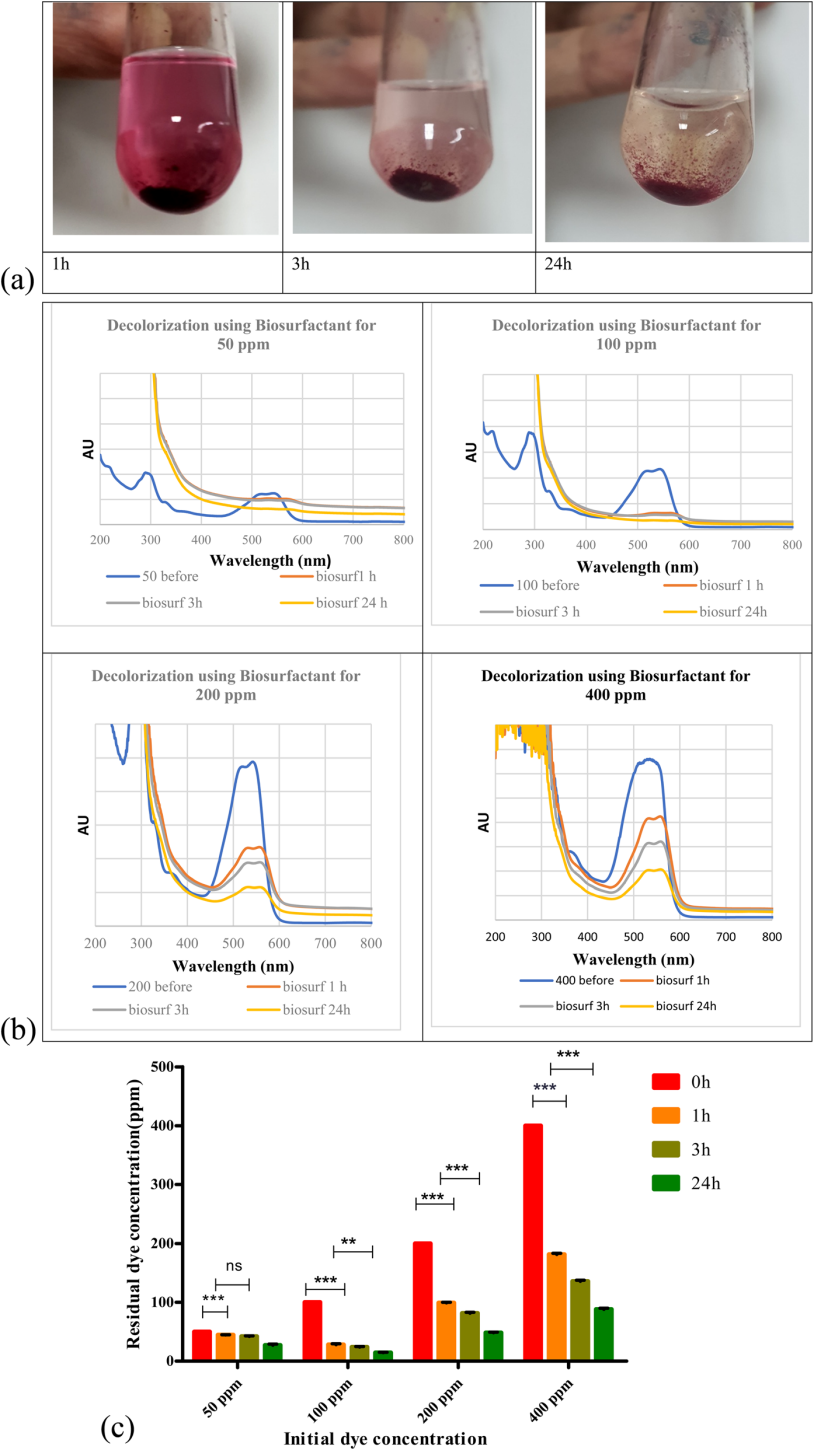
(a) Addition of biosurfactant to 400 ppm nanodye after 1, 3 and 24 h incubation at room temperature. (b) Visible spectra for 50, 100, 200 and 400 ppm nanodye concentration after adding 5 mg mL^−1^ biosurfactant for 1,3 and 24 h. (c) Residual dye concentration for 50, 100, 200 and 400 ppm nanodye after incubation with 5 mg mL^−1^ biosurfactant for 0, 1, 3 and 24 h. Two-way analysis of variance (ANOVA) test followed by Bonferroni's post-test to compare multiple means by row.

Biosurfactant addition to the nanodye revealed flocculation at the bottom of the test tubes over 1, 3 and 24 h as shown in Fig. 4a. Spectra for different dyes before and after incubation with the biosurfactant are shown in Fig. 4b. It is evident that at 50 and 100 ppm nanodye concentrations, the peaks almost vanished, while at 200 and 400 ppm nanodye concentrations, the peaks were visible but with less intensity. This means that dye removal was due to adsorption to the biosurfactant and not degradation. Fig. 4c represents the residual concentrations for each dye concentration after incubation with 5 mg mL^−1^ biosurfactant for 1, 3 and 24 h. The decolorization ranged from 50 to 80% for the different dye concentrations. This result exceeds what^24^ achieved, where they obtained 85% decolorization after 168 h for 100 ppm dye. Rhamnolipid was used to decolorize 100 μg mL^−1^ Direct Orange dye in less than 1 h and under highly acidic conditions.^25^ This means that the lower the dye concentration, the more effective decolorization can be obtained. In the current study, we were concerned about high dye concentrations since the textile dye effluent is concentrated, therefore, the upcoming experiments are performed for 400 ppm dye concentrations as a highly concentrated effluent analogy.

According to ref. 45, biosurfactants act by different mechanisms, they can increase the surface area of water-insoluble substrates by emulsification, or they can also increase the bioavailability of hydrophobic substrates.^46^ mentioned two mechanisms for biopolymer aggregation of particles, either polymer bridging or charge neutralization. When the flocculant has the same charge as the particles, the polymer's chains bring the particles closer together to form flocs. In this case, a cation is usually involved to reduce the effect of the charges and facilitate the adsorption of the particles by the bioflocculant. According to a charge neutralization mechanism, the biopolymer flocculant is expected to reduce the charge density of the particle surface and, as end results, the formation of flocs by reducing the coulombic repulsion forces between particles.

### 3.5. Sequential treatment using electron beam and bioflocculation

Sequential treatment was performed in the following section to test if combining both treatments would lead to higher dye removal. An initial pilot experiment was performed to test the use of electron beam prior to bioflocculant addition and *vice versa*. It is noteworthy to mention that upon testing bioflocculation as the initial step, the residual dye was not affected by electron beam at all (results not shown). Therefore, the sequential experiments were based on the use of electron beam as the initial step.

**Fig. 5:**
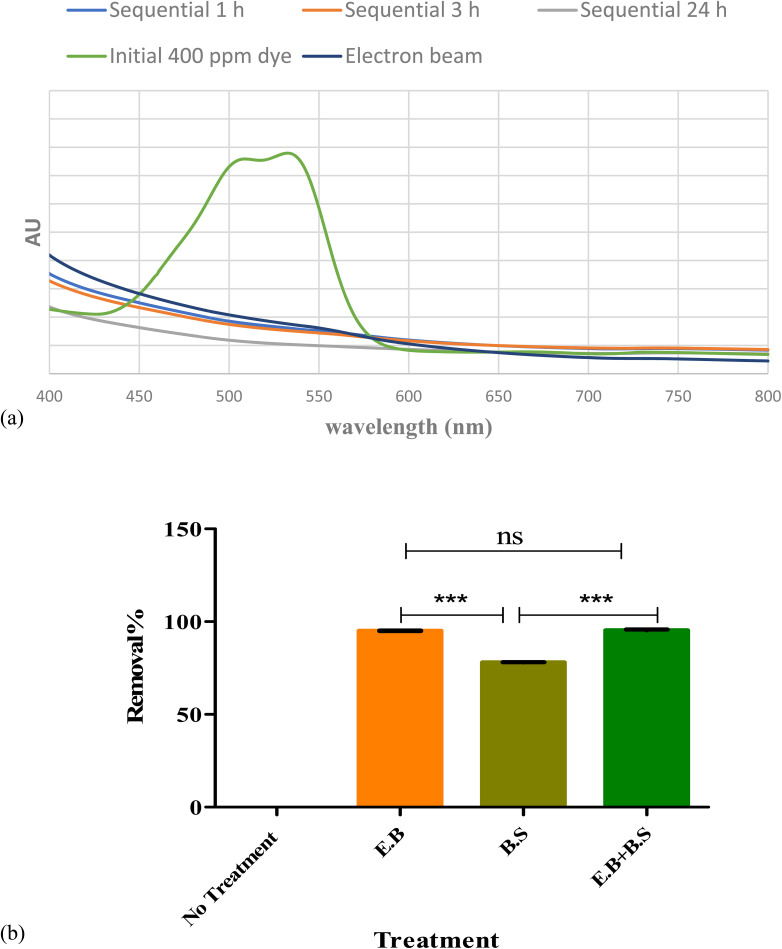
(a) Visible spectrum for 400 ppm before and after using consecutive electron beam (7.5 kGy) and 5 mg mL^−1^ biosurfactant at 1,3 and 24 h incubation. (b) Removal percentages for untreated and different treated 400 ppm nanodye. One-way analysis of variance (ANOVA) test followed by Tukey–Kramer multiple comparison's test.

Fig. 5 a shows the difference in visible spectra of nanodye after 1, 3 and 24 h sequential treatments. It is evident that the peak representing the chromophore disappeared after 1 h and that the color intensity was at its lowest after 24 h. Upon comparing the nanodye removal percentage for single and sequential treatment, about 95% removal can be observed for single and sequential treatment that involves electron beam, while on the other hand, the removal was 78.2% when bioflocculant was used. This result shows that electron beam was more efficient in dye removal in 10 min of the experiment while the use of bioflocculant might have required more optimization of conditions or more time to reach the same result. However, the residual color was observed as mentioned earlier. Removal of this residual brown color would be essential for aesthetic reasons.

As mentioned in Section 3.4, the successful use of biosurfactants in dye decolorization requires further treatment, longer time and is efficient for lower dye concentrations.^47^ reported different dye removal according to the dye chemical class. Their removals reached 80% for direct and disperse dye solution but only 23% for reactive dye solution. It may also be due to oversaturation of bioflocculant with excess dye molecules and consequently decrease in electrostatic force attraction between the molecules. Increasing bioflocculant concentration may result in incomplete dispersion in the solution, as well as a reduction in flocculation activity.^48,49^

**Fig. 6:**
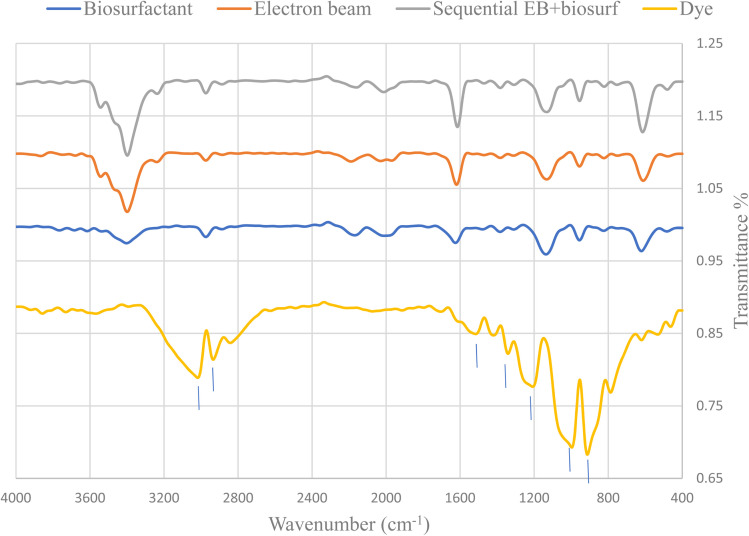
FTIR spectrum of nanodye (400 ppm) before and after 7.5 kGy EB irradiation, bioflocculation and sequential electron beam and bioflocculation.

Fig. 6 represents the changes in functional groups after using single and sequential treatments for 400 ppm nanodye. The results show that for treatments involving electron beam irradiation, the presence of peaks at 3543 and 3398 cm^−1^ indicated hydroxylation of the nanodye, the peak at 1523.7 cm^−1^ disappeared which reflects breaking the –N=N– bond, the disappearance of peaks at 1211.25 and 997 cm^−1^ indicated removal of R-SO_3_^−^.^40^ On the other hand, the appearance of peak at 1614 cm^−1^ represents carbonyl band and the appearance of a secondary amine peak at 625 cm^−1^.^50^ Examining the FTIR for the bioflocculation treated nanodye, the spectrum resembles that for the electron beam exposed samples, but without the appearance of the peaks that indicate hydroxylation. This suggests that the mechanism of degradation of the nanodye for electron beam exposed samples depends primarily on hydroxylation and azo dye bond breakage while that for bioflocculation removal depends on adsorption mainly and degradation as a secondary step. Using sequential treatment resulted in removal of residual brown color usually seen after electron beam irradiation and can be achieved within 1 h. Thus, combining the benefits of both treatment approaches under study. The use of electron beam in degradation of pollutants is more economical than other AOPs. If we consider the time needed for operation, the process can vary from nanoseconds to a few minutes, depending on the content of the sample and the required dose. There are no chemical residues left from the treatment process, it's non-selective. Electron beam is a green technology that doesn't cause any pollution or produce sludge. The use of electron beam before using biological treatment in dyeing wastewater can help in decrease processing time which means reduction in energy for degradation.^51^

### 3.6. Toxicity after separate and sequential treatments of the nanodye

**Fig. 7:**
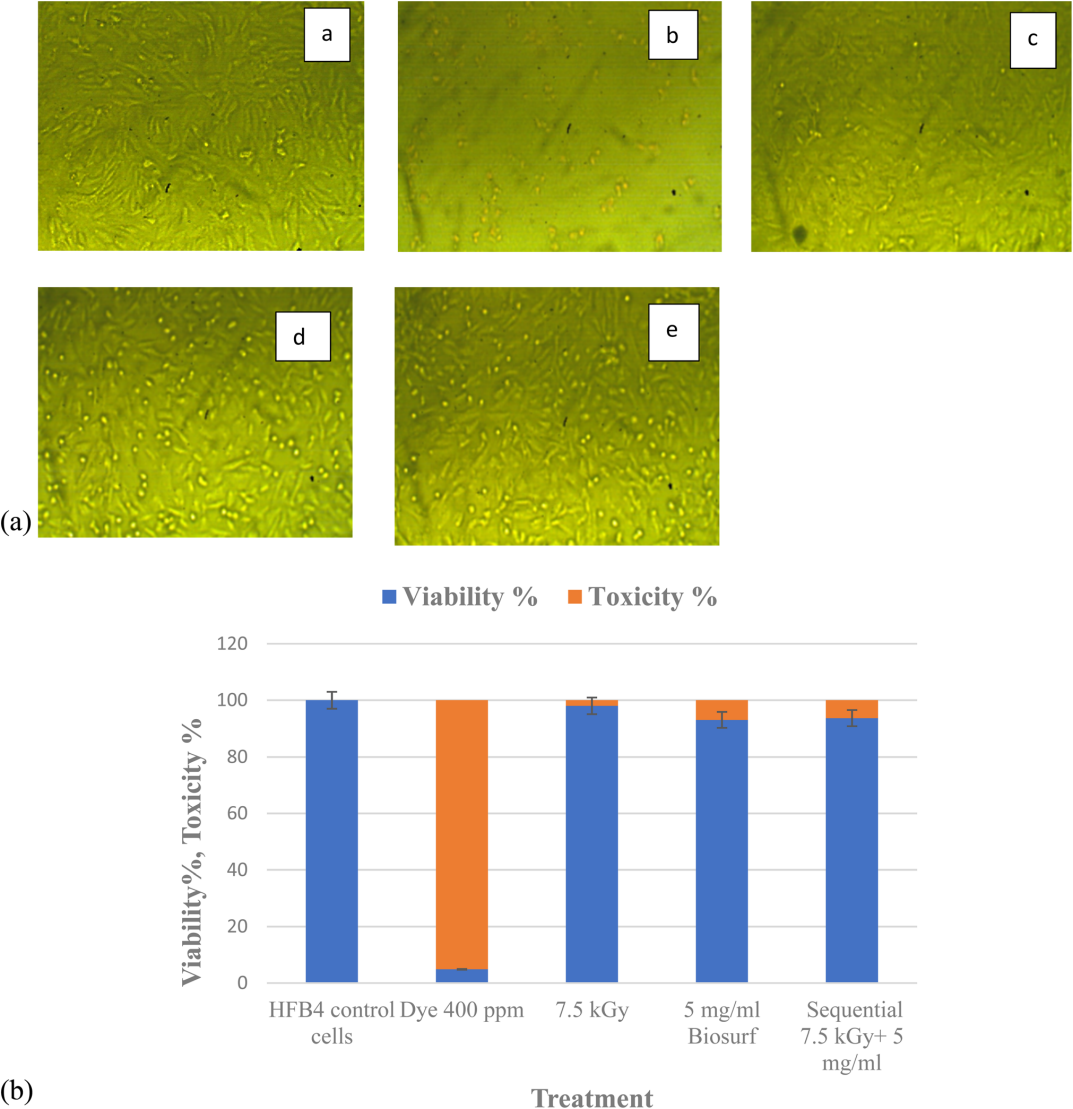
(A) Morphology of HFB4 cells for untreated cells (a) and cells treated with 400 ppm nanodye (b), residual dye after exposure to 7.5 kGy radiation (c), residual dye after treatment with 5 mg mL^−1^ biosurfactant (d) and residual dye after sequential treatment with 7.5 kGy radiation and 5 mg mL^−1^ biosurfactant (e). Images were taken at 10× using light microscope. Scale bar is 1 mm. (B) Viability and toxicity % for HFB4 cells before and after different treatments.

Toxicity evaluation of the by-products post single and sequential treatment was done using MTT assay. Viability of normal dermal cells (HFB4) viability was used as a biomarker that indicates the level of toxicity. This is a crucial step if the treated wastewater is intended for re-use, especially if there will be contact with humans. The results (Fig. 7) show that electron beam irradiated nanodye residual poses the least toxicity of 1.19%, this is followed by the sequential treatment of electron beam and bioflocculation which showed 6.28% and bioflocculation alone as nanodye removal approach posed 6.9% toxicity. Toxicity posed by nanodye before treatment was 95.13%, and that for control (non-treated cells) was 0% toxicity. This result confirms that textile dyes result in loss of cell viability, furthermore, cell cytotoxicity and inflammatory responses can be important sensitive biomarkers that can be used to assess the flocculation/coagulation performance of textile wastewater treatment.^52,53^

## 4. Conclusion

The present investigation provides a practical experimental scenario for testing single and sequential physical (electron beam) and biological (bioflocculants) methods for the treatment of textile nanodyes. The study shows that each approach has its pros and cons. Electron beam provides a fast and versatile approach for dye removal while bioflocculation provides a cheap and up-scalable approach. Therefore, the removal process will depend not only on time and cost but will also depend on the aim of the treatment. If the treated wastewater is needed for cooling purposes, for example, the residual brownish color will not matter, while if treated water will be reused in a new dyeing cycle, then clear water will be needed. The toxicity of the resulting treated wastewater should be taken into consideration as well in case of re-use purposes. If the re-use involves any human-related activity, then electron beam should be used, if no direct contact or minimal need, then bioflocculation can be applied. Either way, a toxicity assay should be performed to make sure that the treatment doesn't pose a threat by affecting cell viability. The results obtained in this study are very promising and can be easily upscaled from lab experiments to reactor tanks.

## Data availability

The data and material will be available upon request.

## Author contributions

Nora M. ElKenawy was responsible for conceptualization, methodology and original draft writing, Ola M. Gomaa was responsible for conceptualization, methodology, review and final editing.

## Conflicts of interest

The authors declare no competing interest.

## Supplementary Material
